# Magnetically Driven Muco-Inert Janus Nanovehicles for Enhanced Mucus Penetration and Cellular Uptake

**DOI:** 10.3390/molecules27217291

**Published:** 2022-10-27

**Authors:** Yue Hao, Shu Bai, Linling Yu, Yan Sun

**Affiliations:** 1 Department of Biochemical Engineering, School of Chemical Engineering and Technology, Tianjin University, Tianjin 300350, China; 2 Key Laboratory of Systems Bioengineering and Frontiers Science Center for Synthetic Biology (Ministry of Education), Tianjin University, Tianjin 300350, China

**Keywords:** mucus penetration, nanovehicle, Janus nanoparticle, magnetically driven, zwitterionic polymer

## Abstract

One of the main challenges of transmucosal drug delivery is that of enabling particles and molecules to move across the mucosal barrier of the mucosal epithelial surface. Inspired by nanovehicles and mucus-penetrating nanoparticles, a magnetically driven, mucus-inert Janus-type nanovehicle (Janus-MMSN-pCB) was fabricated by coating the zwitterionic polymer poly(carboxybetaine methacrylate) (pCB) on the mesoporous silica nanorod, which was grown on one side of superparamagnetic Fe_3_O_4_ nanoparticle using the sol–gel method. X-ray diffraction, transmission electron microscopy, vibrating sample magnetometry, and Fourier infrared spectroscopy were used to characterize the structure and morphology of the nanovehicles, proving the success of each synthesis step. The in vitro cell viability assessment of these composites using Calu-3 cell lines indicates that the nanovehicles are biocompatible in nature. Furthermore, the multiparticle tracking, Transwell^®^ system, and cell imaging experimental results demonstrate that both the modification of pCB and the application of a magnetic field effectively accelerated the diffusion of the nanovehicles in the mucus and improved the endocytosis through Calu-3. The favorable cell uptake performance of Janus-MMSN-pCB in mucus systems with/without magnetic driving proves its potential role in the diagnosis, treatment, and imaging of mucosal-related diseases.

## 1. Introduction

Mucus is a protective barrier with a complex composition [1] that is located in various regions of the body, such as the ocular and nasal surfaces, gastrointestinal tract, cervicovaginal tract, buccal region, and pulmonary airways. Through hydrogen bonding, disulfide bonding, and hydrophobic or electrostatic interactions, mucus effectively traps and promotes the clearance of pathogens and exogenous compounds to protect the mucosal surface [2]. Similarly, the mucus barrier seriously hinders the diffusion and absorption of drugs and other substances to the underlying mucosal epithelium [3,4]. Thus, overcoming the mucosal barrier is a major requirement for the treatment of mucosal diseases, and many strategies have been reported [5,6].

In recent years, mucus-penetrating particles (MPPs) have become a competitive candidate due to their enhanced efficiency in drug delivery by permeating the mucus barrier [7,8,9]. With a hydrophilic and electrically neutral (i.e., muco-inert) surface, as in the case of viruses, and a particle size smaller than the mucus pore size, MPPs can easily penetrate through the mucus layer by reducing the interaction between the particles and mucus, improving the local drug delivery to mucosal epithelial cells [10,11,12]. Coating nanoparticles with poly(ethylene glycol) (PEG) of a high density and low molecular weight is one of the most widely used strategies for mucus penetration. However, PEGylation usually cannot enhance cellular uptake and stimulate the production of neutralizing antibodies [13,14,15,16]. By comparison, zwitterionic polymer coatings can endow nanoparticles with superior hydrophilic and neutral surfaces [17], causing not only improved penetrability in the mucus but also enhanced cellular uptake [18,19]; thus, they have been widely used in the development of transmucosal drug delivery systems. In particular, poly(carboxybetaine methacrylate) (pCB) is a super-hydrophilic, anti-protein adsorption material (with a nonspecific protein adsorption of <5 ng/cm^2^ for undiluted plasma) with excellent biocompatibility and a high performance in terms of the cellular uptake and blood circulation time [20,21,22]. Furthermore, it is easy to modify and derivatize pCB on the surfaces of various materials [23,24,25]. Thus, pCB is a good candidate that may be used to facilitate the mucus permeation of nanoparticles.

Alternatively, nanovehicles with an efficient propulsion provide a new concept for enhancing the ability to overcome the mucus barrier [26]. Nanovehicles are devices that can convert different forms of energy or fuel into kinetic energy and are increasingly used in drug delivery [27], catalysis [28], environmental monitoring [29], and biosensing [30]. Generally, nanovehicles include self-propelled catalytic nanomotors and fuel-free nanorobots powered by biocompatible external energy, such as light, electricity, sound, magnetism, and chemistry energies [31,32,33]. In particular, magnetically driven nanovehicles can be remotely actuated in living organisms by oscillating, rotating, and gradient magnetic fields [34,35,36,37,38], providing new solutions for overcoming the mucus barrier. In particular, the use of gradient magnetic fields is a simple and more accessible method for driving nanovehicles, without the need for complex fabrication techniques and expensive equipment [37].

**Figure 1:**
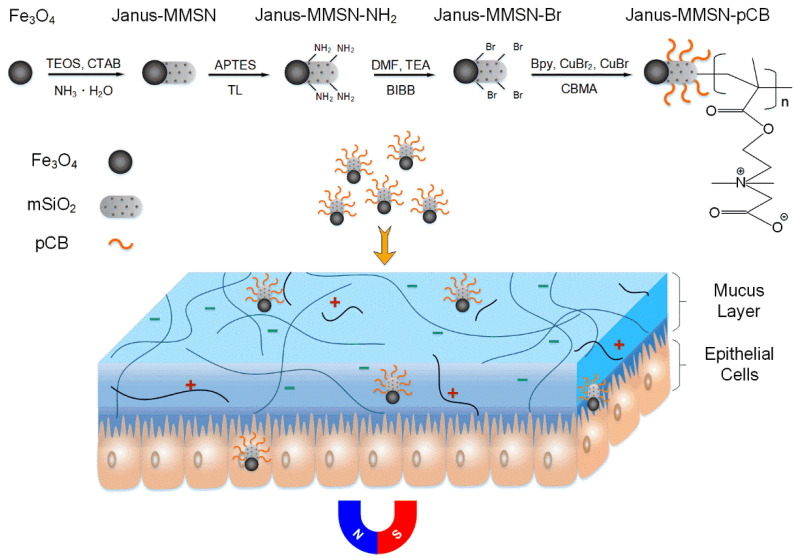
Schematic illustration of the synthesis of magnetically driven, muco-inert Janus-MMSN-pCB nanovehicles. (TEOS: tetraethyl orthosilicate; CTAB: hexadecyl trimethyl bromide ammonium; APTES: 3-aminopropyltriethoxysilane; TL: toluene; DMF: N,N′-dimethylformamide; TEA: triethyl amine; BIBB: α-bromoisobutyryl bromide; Bpy: 2′-bipyridine; CBMA: carboxybetaine methacrylate; pCB: poly(carboxybetaine methacrylate); mSiO_2_: mesoporous SiO_2_).

Hence, a combination of MPP and magnetically driven nanovehicle strategies were explored in this work. In detail, a magnetically driven Janus-type nanovehicle (Janus-MMSN) based on Fe_3_O_4_ nanoparticles and mesoporous silica nanorods was synthesized using a solvothermal method and sol–gel method. By atom transfer radical polymerization (ATRP), the zwitterionic pCB was grafted from the surface of Janus-MMSN to form a magnetically driven, muco-inert nanovehicle (Janus-MMSN-pCB), as shown in Figure 1. The effects of the zwitterionic polymers and magnetic fields on the motion behavior and mass transfer efficiency of the nanovehicles in simulated mucus were explored by in vitro experiments. In addition, using Calu-3 cells, which are mucus-producing lung cancer cells, as a model, the biocompatibility and cellular uptake ability of the nanovehicles were studied. The research illustrates that the magnetically driven, muco-inert nanovehicles (Janus-MMSN-pCB) both enhanced the mucus penetration and mucosal epithelial cell endocytosis and can facilitate the development of mucosal carriers using a nanovehicle-based strategy.

## 2. Results and Discussion

### 2.1. Preparation and Characterizations of the Nanovehicles

**Figure 2:**
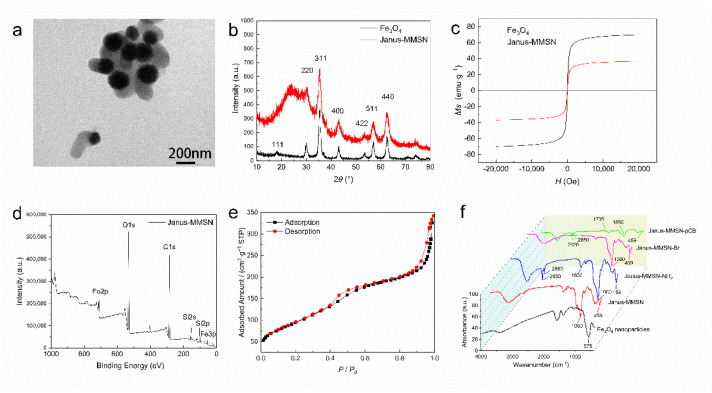
Preparation and characterization of Fe_3_O_4_ nanoparticles and Janus nanovehicles. (**a**) TEM images of Janus-MMSN. (**b**) XRD spectra of Fe_3_O_4_ nanoparticles and Janus-MMSN. (**c**) Magnetic hysteresis loops of Fe_3_O_4_ nanoparticles and Janus-MMSN. (**d**) XPS spectra of Janus-MMSN. (**e**) Nitrogen adsorption/desorption isotherms of Janus-MMSN. (**f**) FT-IR spectra of Fe_3_O_4_ nanoparticles and nanovehicles.

Representative TEM images of the Fe_3_O_4_ nanoparticles and Janus-MMSN are shown in Figure 2a and Figure S1a, respectively. As shown in Figure S3a, the Fe_3_O_4_ nanoparticles had a uniform size of ≈190 nm and exhibited a good dispersion. Janus-MMSN showed a typical asymmetric Janus structure, consisting of Fe_3_O_4_ nanoparticles (dark color) and silica nanorods (light color), with an aspect ratio of 2:1 (Figure 2a).

The XRD spectra of the Fe_3_O_4_ nanoparticles and Janus-MMSN are given in Figure 2b. It is clear, in Figure 2b, that both the Fe_3_O_4_ nanoparticles and Janus-MMSN showed diffraction peaks at 2θ of 30°, 35°, 43°, 53°, 57°, and 63°, indicating that these Janus-MMSNs contained Fe_3_O_4_. In addition, the broad peaks near 2θ of 25° (Figure 2b, black line) confirmed the existence of amorphous silica on Janus-MMSN. The magnetic properties of the Fe_3_O_4_ nanoparticles and Janus-MMSN were examined using a vibrating sample magnetometer (VSM). Since their particle size is much larger than 10 nm, and nearly no remanence or coercivity was detected at 300 K, this indicates that they are soft ferromagnets with a very low coercive force (Hc), which allows them to avoid aggregation when no magnetic field is applied [39]. Because of the nonmagnetic SiO_2_ on its surface, the saturation magnetization of Janus-MMSN (36.80 emu·g^−1^) is lower than that of the Fe_3_O_4_ nanoparticles (70.13 emu·g^−1^). However, Janus-MMSN still possesses excellent magnetic responsivity, achieving the general requirements for magnetic materials [40].

The XPS spectrum of Janus-MMSN (Figure 2d) showed sharp peaks at 102.5 eV and 154.0 eV, which are attributed to the characteristic peaks of Si 2p and Si 2s of SiO_2_, respectively [41]. Moreover, the characteristic peaks of iron were observed at approximately 710.5 eV, indicating that the Fe_3_O_4_ nanoparticles are not completely covered by SiO_2_. The high-resolution spectrum of Fe 2p (Figure S3b) splits into two peaks at 710.6 eV and 724.6 eV, which are in good agreement with the known values of the Fe 2p3/2 and Fe 2p1/2 oxidation states [42]. These results further confirmed that Janus-MMSN contained both Fe_3_O_4_ and amorphous silica.

Figure 2e shows the structural parameters of Janus-MMSN obtained through nitrogen adsorption/desorption. As shown in Figure 2e, Janus-MMSN exhibited type IV isotherms, which are characteristic of mesoporous materials [43]. Using the Barrett–Joyner–Halenda (BJH) method, the calculated pore diameter was approximately 3 nm (Figure S3c). In addition, the Brunauer–Emmett–Teller (BET) surface area and cumulative pore volume of Janus-MMSN were as high as 338.3 m^2^·g^−1^ and 0.5295 cm^3^·g^−1^, respectively. This is equivalent to the pore volume of the worm-like mesoporous SiO_2_ synthesized by Zhang et al. [44], which provided enough sites for small-molecule drugs. Additionally, the carboxyl groups on pCB can be used for immobilizing many drugs, including small molecules and proteins [45,46]. Hence, the nanovehicles are good candidates for the loading of different molecules. Herein, the fluorescein isothiocyanate (FITC) was used as the loading molecule to reveal the potential of the applications of the nanovehicles in the diagnosis, treatment, and imaging of mucosal-related diseases.

The FTIR spectra of all the nanoparticles are given in Figure 2f. It is clear that the peaks at 575 cm^−1^ related to Fe-O stretching can be observed in Figure 2f, indicating the existence of Fe_3_O_4_ in all the nanoparticles. The peaks at 459 cm^−1^ and 1080 cm^−1^ (Figure 2f, red line) correspond to Si-O asymmetrical bending and Si-O-Si asymmetrical stretching vibrations, respectively, indicating that the mesoporous silica rods were successfully grown on the Fe_3_O_4_ nanoparticles. After amination, Janus-MMSN-NH_2_ showed the bending vibration of N-H at 1630 cm^−1^ and the antisymmetric and symmetric stretching vibrations of -CH2- at 2850 cm^−1^ and 2920 cm^−1^ (Figure 2f, blue line), suggesting that Janus-MMSN was successfully modified by APTES. There was no carbonyl peak of BIBB on Janus-MMSN-Br, which is consistent with the spectrum obtained by Lee et al., who modified BIBB on filter paper [47]. The green line in Figure 2e shows a new stretching vibration of C=O at 1735 cm^−1^, demonstrating that pCB was successfully grafted from Janus-MMSN. Meanwhile, these modifications were further confirmed by determining the dynamic light scattering (DLS) and zeta potential (Table S1 and Figure S4). Compared with Janus-MMSN and Janus-MMSN-NH_2_, the average particle size of Janus-MMSN-pCB was slightly larger, and the zeta potential changed to 7.9 ± 0.33 mV, indicating the successful grafting of the pCB. Moreover, the 100 nm increase in the average particle size of Janus-MMSN-pCB compared to Janus-MMSN-NH_2_ indicated that the thickness of the pCB grafting layer (average length of the polymer grafts) was about 100 nm in the hydrated state.

In short, each step of the synthesis of Janus-MMSN-pCB was successful, suggesting that Janus-MMSN-pCB possesses excellent magnetic responsivity, with a high drug loading capacity. Furthermore, considering that both Janus-MMSN and Janus-MMSN-pCB have a Janus-type structure with a magnetic head and a non-magnetic body, they could move under not only gradient magnetic fields but also rotating magnetic fields, near-infrared radiation, and ultrasonic fields [30,48,49]. In this work, we explored their behaviors with the application of a gradient magnetic field of 2 T/m with a maximum magnetic strength of 20 mT. 

### 2.2. Mucus Permeation Analysis

#### 2.2.1. Multiple-Particle Tracking

**Figure 3:**
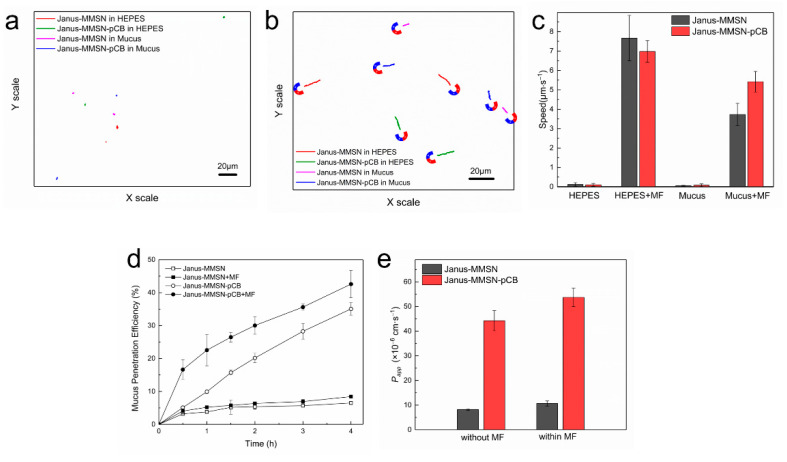
Mucus permeation analysis of Janus-MMSN and Janus-MMSN-pCB. Trajectories of Janus-MMSN and Janus-MMSN-pCB in HEPES (pH 7.4) and simulated mucus, without (**a**) or within (**b**) magnetic fields (t = 3 s, with a magnetic field strength of 20 mT and a gradient of 2 T/m). The average speed (**c**) of Janus-MMSN and Janus-MMSN-pCB in HEPES (pH 7.4) and simulated mucus. Permeation efficiency (**d**) and apparent permeability coefficient (P_app_) (**e**) of Janus-MMSN and Janus-MMSN-pCB penetrating mucus in a Transwell^®^ system.

Figure 3a,b shows the trajectories of Janus-MMSN and Janus-MMSN-pCB in 3 s, without or within magnetic fields, respectively. It is clear that in the absence of magnetic fields, both Janus-MMSN and Janus-MMSN-pCB exhibited typical Brownian motions in the HEPES buffer and mucus, and their trajectories resembled dots (Figure 3a). When a gradient magnetic field (2 T/m) with a fixed direction was applied, both Janus-MMSN and Janus-MMSN-pCB moved in the direction of the magnetic fields in the HEPES buffer and simulated mucus, showing a higher speed and longer trajectories (Figure 3b). Additionally, we proved that these nanovehicles can move in any direction with a magnet.

The trajectories of Janus-MMSN and Janus-MMSN-pCB in the HEPES buffer and simulated mucus were used to calculate the average speed, and the results are shown in Figure 3c. Without MF treatment, Janus-MMSN and Janus-MMSN-pCB diffused randomly in the HEPES buffer and mucus, and the average velocity of Janus-MMSN-pCB in HEPES was only 0.10 ± 0.07 μm·s^−1^ (≈0.26 body lengths·s^−1^). However, under the application of a gradient MF, Janus-MMSN-pCB was able to reach a speed of 6.98 ± 0.56 μm·s^−1^ (≈18.17 body lengths·s^−1^) in HEPES, which was about 70 times faster than that without the MF treatment. The results proved the necessity of the MF treatment for increasing the motion speed of the nanovehicles.

In HEPES buffer, the motion behavior of Janus-MMSN was similar to that of Janus-MMSN-pCB, whether within or without the magnetic fields (Figure 3a,b). However, in mucus, the average speeds of the two nanovehicles under a gradient magnetic field were significantly different. As shown in Figure 3d, in the gradient magnetic fields, the average speeds of Janus-MMSN and Janus-MMSN-pCB in the mucus dropped to 3.73 ± 0.57 μm·s^−1^ (≈9.70 body lengths·s^−1^) and 5.42 ± 0.53 μm·s^−1^ (≈14.11 body lengths·s^−1^), respectively. That is, the two nanovehicles decreased by 56.06% and 22.35%, respectively, compared with the results for the HEPES buffer, demonstrating that the mucus had a significant blocking effect on the movement of the nanovehicles. In particular, the average speed of Janus-MMSN-pCB in the mucus reached as high as 5.42 ± 0.53 μm·s^−1^ (≈14.11 body lengths·s^−1^), which was about 1.5 times that of Janus-MMSN, proving that the modification with the zwitterionic polymer prevented Janus-MMSN-pCB from binding with the mucus and increased the motion speed. This is due to the fact that the super-hydrophilicity and electric neutrality of the pCB provided the muco-inert properties, and the hydrophilic and electrically neutral surface of the nanovehicles coated with pCB greatly weakened the interactions between the nanovehicles and the components in the mucus. Under magnetic propulsion, the average speed of Janus-MMSN-pCB in mucus was comparable to the reported research findings (2.6 ± 0.8 μm·s^−1^, ≈1.04 body length·s^−1^, at a frequency of 30 Hz and magnetic field strength of 10 mT) [26], showing its excellent magnetic-driven motion ability in mucus.

Though most mucus barriers in the human body have irregular forms and directions, it has been proven that the particles with enhanced motion speeds in mucus greatly increase the mucus penetration [41,43]. Furthermore, the magnetic properties of the nanoparticles add an extra level of control in terms of the traveling speed and direction. For example, nanomagnetosols, in combination with a target-directed magnetic gradient field, achieved the targeted aerosol delivery to the lungs of mice [50]. Moreover, the use of magnetic nanoparticles with improved mucus targeting, achieved by the application of a magnetic field, has been reported in vivo and clinical applications [51,52,53,54]. Therefore, in practice, the nanovehicles can be formed into aerosols or suppositories, treating the patient’s mucosal sites with the guidance of an external magnetic field. The magnetic field can be provided by electromagnets or permanent magnets, positioned on the exterior surface of the patient’s body and controlled by adjusting the strength and frequency of the magnetic field.

#### 2.2.2. Mucus Diffusion Analysis

Then, the Transwell^®^ chamber was used to establish an in vitro mucus layer model to evaluate the penetration ability of Janus-MMSN and Janus-MMSN-pCB through a mucus layer [18,55,56,57]. The polycarbonate membrane (3.0 μm) between the donor chamber and the receptor chamber is impermeable for the mucus but allows the nanovehicles to permeate it [51]. The penetration efficiency and P_app_ values of Janus-MMSN and Janus-MMSN-pCB in mucus with/without a magnetic field drive are summarized in Figure 3d,e. As shown in Figure 3d, in the absence of magnetic fields, the mucus penetration efficiency of Janus-MMSN-pCB at 4 h was 35.05 ± 1.90%, which was significantly higher than that of Janus-MMSN (6.51 ± 0.38%). The results further proved that the muco-inert surface of the pCB coating effectively blocked the adhesive interactions between the nanovehicles and mucus components. 

Driven by an external magnetic field of 20 mT with a gradient of 2 T/m, Janus-MMSN and Janus-MMSN-pCB penetrated the mucus more efficiently (8.44 ± 0.09% and 42.59 ± 4.17%, at 4 h), as shown in Figure 3d, proving that the application of the magnetic field also promoted their diffusion in the mucus. However, it is worth noting that at 0.5 h, the mucus penetration efficiency of Janus-MSN-pCB driven by a magnetic field (16.63 ± 2.96%) was significantly higher than that without a magnetic field (5.13 ± 0.42%). Then, the mucus penetration efficiency increased to 42.59 ± 4.17% and 35.05 ± 1.90% at 4 h, respectively, showing a weakened improvement under the influence of the magnetic field. This weakened improvement may be due to the assembly of the nanovehicles into micrometer-sized aggregates after the application a magnetic field for a long time [58], which cannot easily pass through the polycarbonate membrane with a pore size of 3.0 µm. The results indicated that the improvement yielded by the magnetic fields is evident for a short time, but it is not as large as that of the pCB coating for a long time. 

As shown in Figure 3e, the P_app_ value of Janus-MMSN-pCB under the application of the magnetic fields was significantly higher than that of Janus-MMSN (53.70 × 10^−6^ cm·s^−1^ vs. 10.65 × 10^−6^ cm·s^−1^). Similarly, without magnetic fields, Janus-MMSN-pCB showed an evidently larger P_app_ value than Janus-MMSN (44.24 × 10^−6^ cm·s^−1^ vs. 8.22 × 10^−6^ cm·s^−1^). Furthermore, whether with or without the magnetic fields, the P_app_ value of Janus-MMSN-pCB was higher than that of the MPPs reported previously (12 × 10^−6^ cm·s^−1^ ~ 30 × 10^−6^ cm·s^−1^), which were also modified with zwitterionic polymers [18,43,59]. These results revealed that Janus-MMSN-pCB had an excellent mucus penetrability. In short, Janus-MMSN-pCB possesses an excellent mucus penetrability and magnetic-driven motion ability.

### 2.3. In Vitro Cytotoxicity and Cellular Uptake

**Figure 4:**
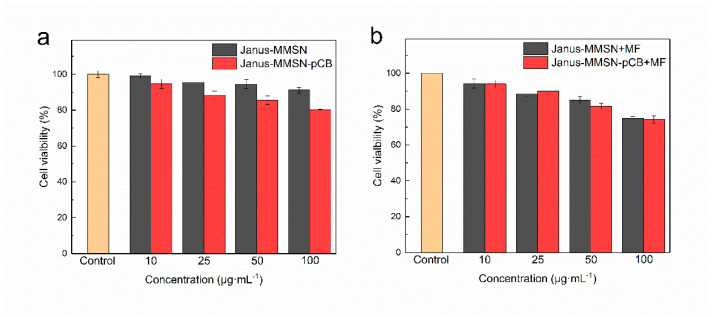
In vitro cytotoxicity of Janus-MMSN and Janus-MMSN-pCB to Calu-3 cells without a magnetic field (**a**) or with a magnetic field (**b**). (The magnetic field strength is 20 mT, with a gradient of 2 T/m).

An MTT assay was performed to evaluate the cytotoxicity of Janus-MMSN and Janus-MMSN-pCB on the Calu-3 cells, which express mucin genes and secrete mucus, and the results are summarized in Figure 4. As shown in Figure 4a, in the absence of magnetic fields, the viabilities of both Janus-MMSN and Janus-MMSN-pCB were all higher than 80%, suggesting that the nanovehicles had no significant cellular toxicity at concentrations ranging from 10 μg·mL^−1^ to 100 μg·mL^−1^. With the magnetic field treatment, the cell viabilities decreased (Figure 4b). The decrease in viability may be due to the magnetic fields, which can drive the nanovehicle to penetrate the mucus layer so as to enter or attach to the Calu-3 cells more quickly (as discussed in Section 3.2), affecting the growth of the cells. It is clear, in Figure 4b, that the cell viabilities of the two nanovehicles were still higher than 70%, despite their incubation with the highest concentration of 100 μg·mL^−1^. The results demonstrated that both Janus-MMSN and Janus-MMSN-pCB had low cytotoxicity in regard to the Calu-3 cells.

**Figure 5:**
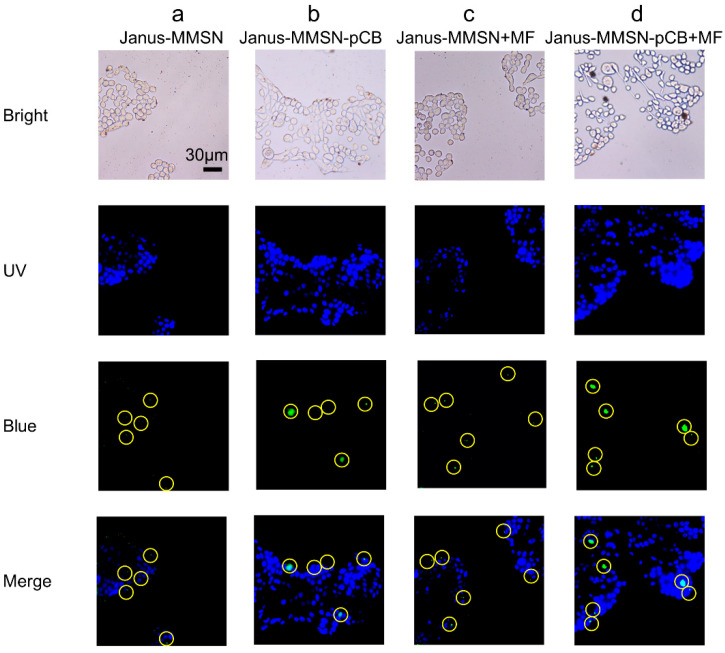
Cell imaging of Calu-3 cells cocultured with Janus-MMSN and Janus-MMSN-pCB for 4 h. (**a**) Janus-MMSN without magnetic driving; (**b**) Janus-MMSN-pCB without magnetic driving; (**c**) Janus-MMSN with magnetic driving; (**d**) Janus-MMSN-pCB with magnetic driving. Yellow dashed circles are used to highlight the green dots in these cells.

To further study the uptake of Janus-MMSN and Janus-MMSN-pCB in the Calu-3 cells, a fluorescent inverted microscope was used to observe the FITC-labeled nanovehicles shown by the green color in the cell, which has been used to prove a higher cellular uptake performance in many reports [43,60]. Figure 5 shows the fluorescent images of Janus-MMSN and Janus-MMSN-pCB in the Calu-3 cells, and the overlap between the green (FITC on the nanovehicle) and blue colors (cell nuclei stained with Hoechst 33258) indicated the uptake of Janus-MMSN and Janus-MMSN-pCB into the cell by endocytosis. To clearly demonstrate endocytosis, yellow dashed circles are used to highlight the green dots in Figure 5. The large green dots are the aggregates of the nanovehicles caused by the introduction of magnetic field, but there are also many small green dots in the merged images in Figure 5. Compared with Janus-MMSN (Figure 5a,c), the uptake amounts of Janus-MMSN-pCB (Figure 5b,d) were significantly higher, regardless of whether the magnetic fields were applied. This proved that zwitterionic polymer coatings can increase cellular uptake, as described in previous reports [18,19]. In addition, the nanovehicles treated with magnetic fields (Figure 5c,d) showed a higher cellular uptake than the nanovehicles untreated with magnetic fields (Figure 5a,b) because of the magnetic-driven ability of Janus-MMSN and Janus-MMSN-pCB, as discussed in Section 3.2. Notably, although both Janus-MMSN and Janus-MMSN-pCB could efficiently enter the Calu-3 cells under the drive of magnetic fields, Janus-MMSN-pCB exhibited a much better cellular uptake. These results revealed that Janus-MMSN-pCB exhibited an excellent performance in its cellular uptake with a magnetic field treatment. In short, Janus-MMSN-pCB possesses a low cytotoxicity and enhanced cellular uptake.

In further studies, the stability and drug delivery performance of the nanovehicle system will be investigated based on the environment-responsive bonds (e.g., hydrazone bond, imine bond, and disulfide bond) [61,62,63]. In particular, the hydrazone bond, which is the only acid-sensitive group currently approved by the FDA for controlled drug release systems [64], will be used to release the cargo at the delivery point, and the drug release mechanism of the nanovehicles will be systematically analyzed.

## 3. Materials and Methods

### 3.1. Materials and Cells

Tetraethyl orthosilicate (TEOS) and sodium acetate (NaAc) were purchased from Shanghai Aladdin. Iron chloride hexahydrate (FeCl_3_·6H_2_O), ethylene glycol (EG), hexadecyl trimethyl bromide ammonium (CTAB), 3-aminopropyltriethoxysilane (APTES), β-propiolactone, 2-(dimethylamino)ethyl methacrylate (DMAEMA), α-bromoisobutyryl bromide (BIBB), fluorescein isothiocyanate (FITC), and thiazole blue (MTT) were purchased from Sigma-Aldrich. Toluene, anhydrous ether, anhydrous acetone, N,N′-dimethylformamide (DMF), and triethyl amine (TEA) were purchased from Tianjin Jiangtian Chemical Technology Co., Ltd. Porcine gastric mucin (type II) and lecithin were purchased from Shanghai Yuanye Biotechnology Co., Ltd., (Shanghai, China).

The mucus-producing cells used in the experiment, the Calu-3 cells, were purchased from Beijing Dingguo Biotechnology Co., Ltd., (Beijing, China). Minimum essential medium (MEM) containing 10% fetal bovine serum (FBS), 1% penicillin, and streptomycin (100 μg·mL^−1^) was used to culture the cells. The cells were cultured in flasks and incubated at 37 °C with 5% CO_2_.

The model mucus was prepared according to the methods of other permeability studies [65,66,67]. Briefly, type II porcine gastric mucin (60 mg·mL^−1^), lecithin (3.2 mg·mL^−1^), and BSA (32 mg·mL^−1^) were dispersed in 20 mM HEPES (pH 7.4, containing 5 mM NaCl) for 48 h in a thermostatic shaker at 4 °C. Although commercial porcine gastric mucin does not form an ideal mucus gel, reconstituted mucus derived from porcine gastric mucin has been found to have a physiologically relevant composition and rheological properties that mimic the cystic fibrosis sputum model [65,66,67]. Moreover, nanoparticles designed as drug and/or gene carriers were entrapped in reconstituted mucus through mucin interactions and, subsequently, their transport was hindered [35,43,59]. These results proved that the reconstituted mucus derived from pig gastric mucin works well as a mucus barrier against the foreign particles.

### 3.2. Synthesis of Magnetic Mesoporous Janus Nanovehicles (Janus-MMSN)

Monodisperse magnetic Fe_3_O_4_ nanoparticles were prepared using a solvothermal method [68]. FeCl_3_·6H_2_O (2.16 g, 8 mmol) and sodium citrate (NaCit) (0.40 g, 1.34 mmol) were dispersed in EG (40 mL). After dissolving, NaAc (2.4 g, 29.2 mmol) was added to the system and stirred for 30 min. The solution was transferred to a 100 mL polytetrafluoroethylene-lined stainless-steel autoclave and subjected to 200 °C for 8 h. After the reaction, the Fe_3_O_4_ nanoparticles were washed with ethanol and deionized water 3 to 5 times.

Then, Janus-MMSN was prepared using a sol–gel process [69]. An Fe_3_O_4_ nanoparticle suspension (5.0 mL of 9.0 mg·mL^−1^) was added to CTAB solution (50.0 mL of 5.0 mg·mL^−1^), followed by vigorous ultrasonic treatment for 30 min. Afterward, the mixture was mechanically stirred at 40 °C in an oil bath, and ammonia hydroxide (2.50 mL) was added to the solution, followed by tetraethyl orthosilicate (0.15 mL). After 20 min, ethanol (20.0 mL) was added to terminate the reaction. In the presence of a magnetic field, the product was separated and thoroughly washed three times with ethanol and DI water. Finally, the template was removed by refluxing the samples in an ethanol solution of ammonium nitrate.

### 3.3. Preparation of Muco-Inert Nanovehicles (Janus-MMSN-pCB)

The Janus-MMSN-pCB was prepared using surface-initiated ATRP with BIBB as the initiator and CuBr/2,2′-bipyridine (Bpy) as the catalyst [47,70]. First, Janus-MMSN was functionalized with APTES to obtain an amino group of Janus-MMSN (Janus-MMSN-NH_2_). Janus-MMSN (0.20 g) was dispersed in toluene (40.0 mL), followed by the addition of APTES (4.0 mL). The mixture was stirred and refluxed at 80 °C for 12 h. Then, Janus-MMSN-NH_2_ was washed with ethanol in the presence of magnetic fields.

Thereafter, Janus-MMSN-NH_2_ was immobilized with ATRP initiators (BIBB). Janus-MMSN-NH_2_ (0.2 g) was dispersed in DMF (50 mL), and triethylamine (2.4 mL) was then added. After the dispersion, it was immersed in an ice bath for 10 min, and a solution of BIBB (2 mL) in DMF (5 mL) was added dropwise. The mixture was stirred at 0 °C for 2 h and then continuously stirred for another 18 h at room temperature. Janus-MMSN-Br was washed with DMF, toluene, and DI water 3 times.

Finally, pCB was grafted from the surface of Janus-MMSN-Br by ATRP using the CuBr/Bpy system as a catalyst with a carboxybetaine methacrylate (CBMA) monomer, which was synthesized as reported previously [47]. The 1HNMR spectrum is shown in the supporting information (Figure S1). Briefly, Janus-MMSN-Br (0.1 g) was dispersed in 50% DMF (20 mL), and then CBMA monomer (2.27 g, 10.0 mmol), CuBr2 (4.46 mg, 0.02 mmol), and Bpy (31.24 mg, 0.2 mmol) were added in sequence. The dispersion was stirred under N_2_ for 30 min. Then, CuBr (14.35 mg, 0.1 mmol) was quickly added, and the reaction was stirred for 3 h in a N_2_ atmosphere. Finally, the mixture was exposed to air so as to terminate the reaction. After the suspension turned blue, Janu-MMSN-pCB was washed with EDTA solution (0.1 mol·L^−1^) until the supernatant was colorless and then washed with ethanol and DI water 3 times.

### 3.4. Characterizations of the Nanoparticles

The morphologies of the nanoparticles were observed by transmission electron microscopy (TEM, JEM-2100F, JEOL, Tokyo, Japan). A chemisorption–physisorption analyzer (TriStar II3020, Micrometrics, Norcross, GA, USA) was used to measure the structural parameters of Janus-MMSN by nitrogen adsorption/desorption analysis at 77 K. The Brunauer–Emmett–Teller (BET) method was applied to evaluate the specific surface area of the samples, and the Barrett–Joyner–Halenda (BJH) method was applied to calculate the pore size distributions. Fourier transform infrared (FTIR) spectra were collected using an IRPrestige-21 Fourier spectrophotometer with KBr pellets (Shimadzu, Tokyo, Japan). MiniFlex600 powder X-ray diffraction (XRD, Rigaku, Tokyo, Japan) patterns were recorded using Cu-Kα radiation (λ = 1.54056 Å, (0.02°·min^−1^ in the 10–80° range). The magnetic properties of the nanoparticles were examined using a Lake Shore 7404 vibrating-sample magnetometer (USA). ESCALAB 250XI X-ray photoelectron spectroscopy (XPS, Thermo Fisher, Waltham, MA, USA) was used to determine the surface composition of the nanoparticles. The dynamic light scattering (DLS) and zeta potential measurements were conducted using a Zetasizer Nano instrument (Malvern Instruments, Malvern, UK) to detect the size, particle size distribution (polydispersity index, PDI), and zeta potential of the nanoparticles.

### 3.5. Multiparticle Tracking of the Nanovehicles

The kinetic behavior of the magnetically driven nanovehicles was investigated using a multiparticle tracking (MPT) method based on a Nikon TE2000-U inverted fluorescence microscope and a CCD camera [26,58,71]. A total of 10 µL of the FITC-labeled nanovehicles (diluted with HEPES to 1.0 mg·mL^−1^) were mixed with HEPES or mucus (90 µL), respectively, by ultrasonication and placed in a Petri dish after standing for 3 min to rule out the possibility that they were advected by flows. Moreover, an LH10-3 electromagnet consisting of four coils (Beijing Blue Ocean Scientific Instruments Co., Ltd., Beijing, China), as shown in Figure S2, was used to generate a gradient magnetic field with a maximum magnetic induction of 20 mT and a magnetic gradient of 2 T/m, which was controlled by a power supply with editable signals [20,37,72]. The external magnetic field was used to propel the nanovehicles, and the nanovehicles were tracked for a minimum of 10 s. Finally, ImageJ software was employed to analyze the recorded videos and calculate the average velocity of the nanovehicles [73].

### 3.6. Transwell^®^ System Diffusion Analysis

To evaluate the penetrating ability of the nanovehicles through a mucus layer, a 6.5 mm Transwell^®^ system with a 3.0 µm pore polycarbonate membrane insert (Corning, NY, USA) was used [18]. The mucus models were plated onto the inserts at a volume of 20 µL, which was sufficient to cover the membrane. Then, 600 µL HEPES was added into the acceptor compartment. Afterward, 200 µL of the FITC-labeled nanovehicles (diluted with HEPES to 1.0 mg·mL^−1^) was added to the donor side, following a 4 h incubation at 37 °C. At predetermined time points (0.5, 1, 1.5, 2, 3, and 4 h), samples (100 µL) were collected from the receptor side, and an equal volume of prewarmed HEPES was re-added. An Infinite M200 PRO microplate was used to analyze the fluorescence intensity of FITC-labeled nanovehicles at the 485 nm excitation (Tecan, Männedorf, Switzerland). Each experiment was conducted in triplicate and, in this paper, the average value is represented with its standard deviation. The apparent permeability coefficient (*P_app_*, cm·s^−1^) was calculated according to the equation below:

(1) $$P_{app}=\frac{dQ}{dt}\times \frac{1}{A\times C_{0}}$$

where *dQ/dt* is the diffusion rate of FITC-labeled nanovehicles. *C*_0_ is the initial nanovehicle concentration in the donor compartment. *A* is the area (cm^2^) of the membrane.

### 3.7. Cell Viability Study

The effects of the different nanovehicles on the cell viability was tested by an MTT assay [74]. The Calu-3 cells were seeded (8000 cells·well^−1^) on 96-well plates and cultured for 24 h. The cells were then incubated with nanovehicles at different concentrations (in DPBS) for 4 h. The cell viability was analyzed using the MTT assay, and the absorbance of each well was measured at 570 nm using a microplate reader. Finally, the percentage of cell viability was calculated by comparing the absorbance of the treated cells with the negative control.

### 3.8. Cellular Uptake

The cellular uptake of the nanovehicles was observed by inverted fluorescence microscopy [18,60,68]. Calu-3 cells were seeded (5000 cells·well^−1^) on 6-well plates and incubated with the FITC-labeled nanovehicles (50 µg·mL^−1^ in DPBS) for 4 h. Then, the cells were washed 3 times with DPBS and stained with H33258 to enable us to observe the cells and nanovehicles under an inverted fluorescence microscope.

## 4. Conclusions

In this work, a magnetically driven Janus-type nanovehicle (Janus-MMSN) composed of Fe_3_O_4_ nanoparticles and mesoporous silica nanorods was successfully prepared using a solvothermal method and sol–gel method. The ATRP reaction was used to graft the zwitterionic polymer (pCB) onto its surface so as to obtain a mucus-inert, magnetically driven Janus-type nanovehicle (Janus-MMSN-pCB). The electrical neutrality and superhydrophilicity of the zwitterionic polymers (pCB) can effectively prevent Janus-MMSN-pCB from adhering to mucin, and the magnetic-driven ability can respond to an external magnetic field so as to cause the nanovehicles to move quickly through the mucus at a speed 5.42 ± 0.53 μm·s^−1^ (≈14.11 body length·s^−1^). The in vitro mucus penetration experiments showed that the penetration efficiency of Janus-MMSN-pCB under a gradient magnetic field (2 T/m) was as high as 42.35% at 4 h, which was significantly higher than the 8.44% of Janus-MMSN under the same conditions, indicating that pCB grafting was more effective than the application of magnetic fields in improving the diffusion rate of the nanovehicles in the simulated mucus. The in vitro cell experiments showed that Janus-MMSN and Janus-MMSN-pCB had good biocompatibility, and the cellular uptake of Janus-MMSN-pCB was better than that of Janus-MMSN, especially when driven by magnetic fields. The good biocompatibility and superior mucus penetration performance of Janus-MMSN-pCB prove that the combination of MPP and nanovehicles is an effective strategy for constructing efficient carriers for the purpose of transmucosal material delivery and provides a potential carrier for the diagnosis, treatment, and imaging of mucosal-related diseases. 

## Data Availability

Data are available on request from the authors.

## Supplementary Materials

The following supporting information can be downloaded at: https://www.mdpi.com/article/10.3390/molecules27217291/s1, Figure S1: ^1^H NMR spectrum of CBMA; Figure S2: The electromagnet used to generate gradient magnetic fields; Figure S3: Characterization of Fe_3_O_4_ nanoparticles and Janus nanovehicles; Table S1. Zeta potentials and particle size of the nanoparticles; Figure S4: Size distributions of Fe_3_O_4_ nanoparticles and nanovehicles in HEPES (pH 7.4).
