# Supplementary material for: Magnetically Driven Muco-Inert Janus Nanovehicles for Enhanced Mucus Penetration and Cellular Uptake
Source: Molecules. 2022 Oct 27;27(21):7291. doi: 10.3390/molecules27217291 (PMC9654700; doi:10.3390/molecules27217291)
Supplement: Supplementary file 1 [file molecules-27-07291-s001.zip › molecules-1960997-supplementary.pdf]

Supporting Information

Magnetically driven muco-inert Janus nanovehicles for enhanced mucus penetration and cellular uptake

Yue Hao [1], Shu Bai [1,2], Linling Yu [1,2,*] and Yan Sun [1,2,*]

1 Department of Biochemical Engineering, School of Chemical Engineering and Technology, Tianjin University, Tianjin 300350, China
2 Key Laboratory of Systems Bioengineering and Frontiers Science Center for Synthetic Biology (Ministry of Education), Tianjin University, Tianjin 300350, China
* Correspondence: yulinling@tju.edu.cn; ysun@tju.edu.cn

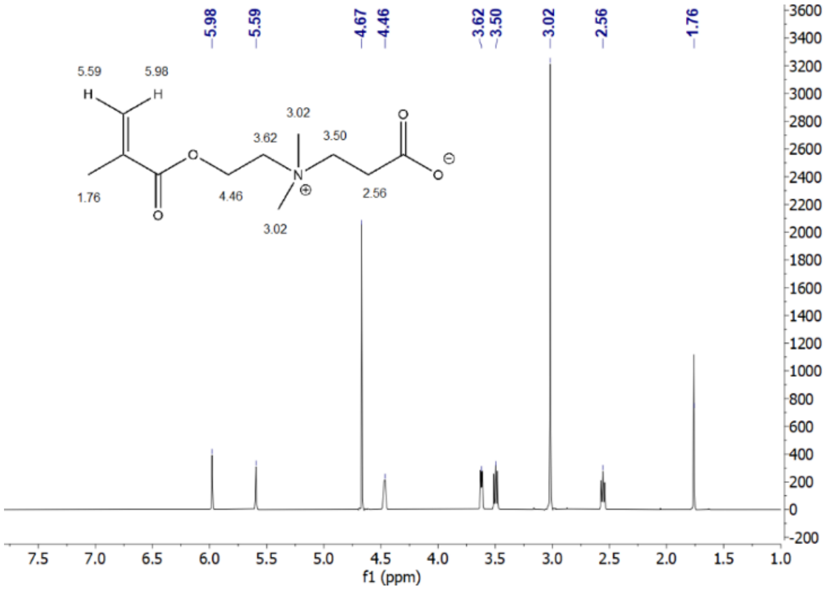

Figure S1. ^{1}H NMR spectrum of CBMA: 1.76 (s, 3H, dCCH3), 2.54 (t, 2H, CH2COO), 3.01 (s, 6H, NCH3), 3.49 (t, 2H, NCH2), 3.61 (t, 2H, CH2N), 4.46 (t, 2H, OCH2), 5.59 (s, 1H, dCH), 6.97 (s, 1H, dCH).

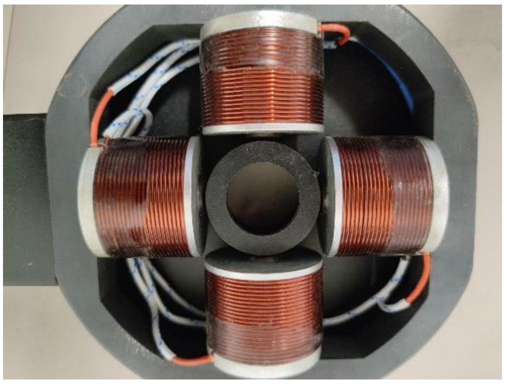

Figure S2. The electromagnet used to generate gradient magnetic fields.

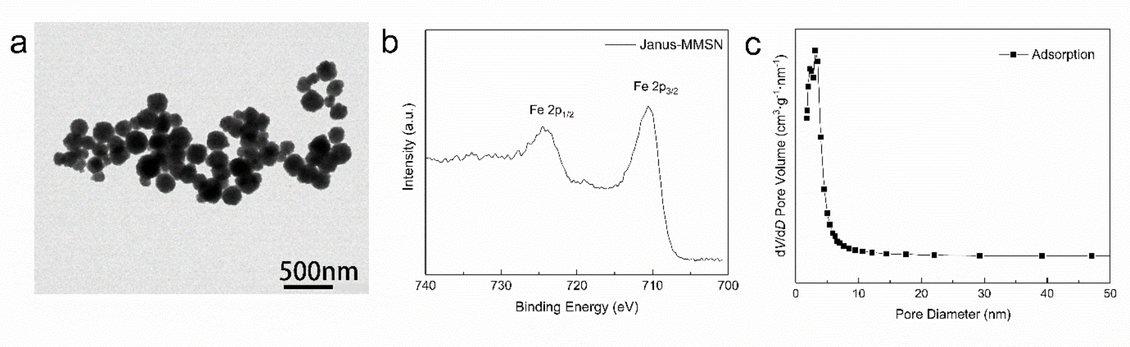

Figure S3. Characterization of Fe_3O_4 nanoparticles and Janus nanovehicles. (a) TEM images of Fe_3O_4 nanoparticles. (b) XPS high-resolution scan of the Fe 2p electron of Janus-MMSN. (c) Pore size distribution of Janus-MMSN.

Table S1. Zeta potentials and particle size of the nanoparticles.

Particle	Zeta potential (mV)	Size (nm)	PDI
Fe_3O_4	-16.4±1.0	196.2±2.1	0.11±0.03
Janu-MMSN	6.4±1.6	590.2±12.4	0.29±0.05
Janus-MMSN-NH_2	21.8±0.4	613.4±12.6	0.25±0.01
Janus-MMSN-pCB	7.9±0.4	719.7±14.1	0.35±0.01

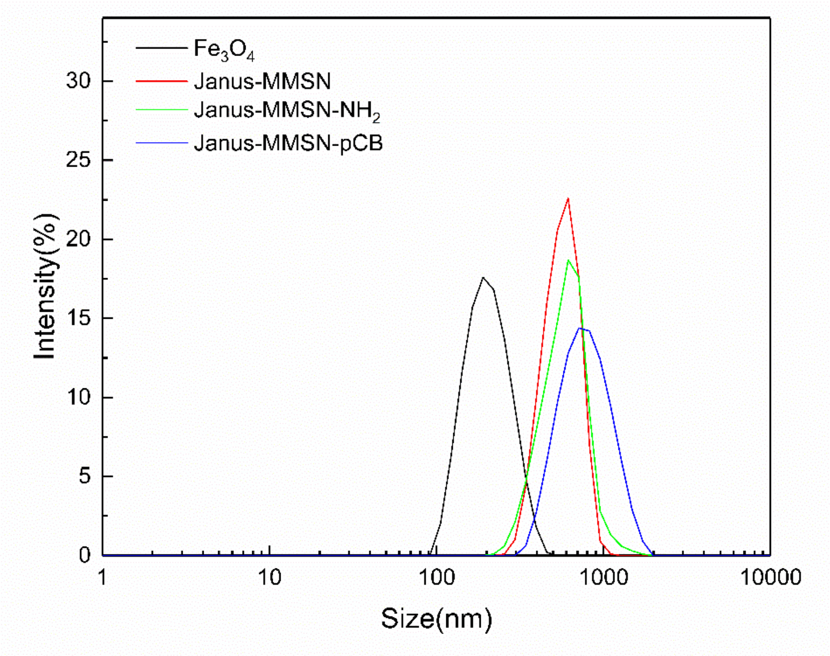

Figure S4. Size distributions of Fe_3O_4 nanoparticles and nanovehicles in HEPES (pH 7.4).
